# Prevalence of stunting and associated factors among preschool children: A community based comparative cross sectional study in Ethiopia

**DOI:** 10.1186/s40795-018-0236-9

**Published:** 2018-07-05

**Authors:** Getnet Berhanu, Solomon Mekonnen, Mekonnen Sisay

**Affiliations:** 1 Albuko district health office, South Wollo, Albuko, Ethiopia; 2 Department of Human Nutrition, Institute of Public Health, College of Medicine and Health Sciences, University of Gondar, Gondar, Ethiopia

**Keywords:** Stunting, preschool, children, associated factors, Ethiopia

## Abstract

**Background:**

The prevalence of under nutrition is very high in developing countries especially in women and under five children. Stunting alone affected an estimated 154.8 million (22.9%) under five children globally in 2016. It is one of the main undernutrition and health problems facing children in Ethiopia. Hence, the aim of the current study was to assess the prevalence and associated factors of stunting among preschool children from food secure and food insecure households in Albuko district, northeast Ethiopia.

**Methods:**

This study was addressed by a community based comparative cross sectional study design which was conducted among preschool children in Albuko district from March to April 2017. Simple random sampling was used to select the five representative kebeles. To reach study participants, systematic sampling technique was utilized. Pretested and structured questionnaire was used to collect data. Height measurement was collected for each child. Anthropometric indicator, height-for-age was determined for children using current WHO growth standards. Adjusted odds ratio (AOR) with its 95 % confidence interval (CI) was computed to assess the strength of the association. To identify the associated factors of stunting, multivariable logistic regression models were built. In the multivariable analysis, variables with a P-value of <0.05 were considered statistically significant.

**Results:**

The overall combined prevalence of stunting among preschool children in the study area was 39.3% [95%CI; 36.3, 42.3%]. A higher stunting prevalence was observed among preschool children from food insecure households [42.8%, 95%CI; 38.4, 47.2%] than food secure ones [35.9%, 95%CI; 31.7, 40.1%]. Having uneducated mothers, large family size, and male sex were common factors significantly associated with stunting in both food secure and insecure households. While child birth order and the amount of water (<40 litters) for use were significantly associated with stunting among preschool children living in food secure households, and lesser child age, lack of extra food during pregnancy/lactation, and low dietary diversity score (DDS below four food groups) were significantly associated with stunting among preschool children from food insecure households.

**Conclusion:**

The present study showed that stunting is an important public health problem among preschool children from both food secure and insecure households in Albuko district. Though productive safety net program (PSNP) is a proven strategy in reducing the burden of childhood undernutrition/stunting, this study showed that there is no significant variation in the magnitude of stunting. However, it does not mean that PSNP interventions are not important in reducing the prevalence of stunting. Therefore, strengthening maternal nutrition, family planning utilization, and maternal education and enhancing dietary diversity, water sanitation and hygiene are critical interventions to reduce the level of stunting among under five children.

## Background

Child mortality in developing countries are mostly due to preventable causes. More than 50 percent of deaths among children below five years old were contributed by malnutrition alone in third world countries [1, 2]. Stunting affected an estimated 22.9% or 154.8 million under five children globally in 2016 and the two continents; Asia and Africa are the home to most children who are stunted and each of them contributed 87 million and 59 million to the burden of stunting among children in the globe respectively [3]. In Africa, one in three children is stunted, and the prevalence (around 40%) to which 14 countries including Ethiopia are home to 80% of stunting among children in the world and contribute 3% of stunting burden globally has remained unchanged in the past twenty years [4, 5].

In Ethiopia, undernutrition remains a serious challenge. As a result, 45 percent of Ethiopian children deaths were associated with undernutrition where its causes are multifaceted [6]. According to Ethiopian Demographic and Health Survey 2016 (EDHS 2016) report, compare to the previous survey report the number of stunted children under five years old showed a decreasing pattern over the last five years from 44% in 2011 to 38% in 2016 but, still 38% of Ethiopian under five children are suffering from stunting of them 18% are severely stunted [7, 8].

Child undernutrition has long-term negative consequences. A child who is undernourished will have repeated episodes of infection, less competent in his education and lower productivity than well-nourished child [9]. And again, a child who is stunted have poor physical growth, cognitive development and low school performance [10, 11]. In addition, the loss in human and economic potential is significant and currently in Ethiopia, undernutrition is responsible for 25 percent of child deaths [12]. According to the cost of hunger analysis in Ethiopia for 2009, Ethiopia has lost 16.5% of her GDP (4.7 billion $US) of 2009 due to undernutrition [12].

As outlined by UNICEF a child from food insecure household will be malnourished [13]. Food security is defined as “Food security exists when all people, at all times, have physical and economic access to sufficient, safe, and nutritious food that meets their dietary needs and food preferences for an active and healthy life” [14]. Being secured in food does not mean that there is an adequate nutrition [15]. It is one of the pillars of improving nutrition status, but doesn’t necessarily mean that children from food secure households are well nourished. Malnutrition is common in many food secure households [16, 17]. A pooled analysis involving children aged 1 week to 59 months in 10 prospective studies in Africa, Asia, and South America (anthropometry cohort pooling) by Ibironke Olofin et al in 2013 showed that infection leads to undernutrition, and all degrees of anthropometric deficits increased the hazards of dying from respiratory tract infections and diarrheal diseases [18].

The causes of undernutrition are multifaceted. Inadequate dietary intake and disease, household food insecurity, poor caring practices, lack of access to basic services, including lack of access to safe water supply, health services (including knowledge and training of health workers); and unhealthy living environment, such as open defecation [19]. In turn, these causes are influenced by ‘economic, political and social conditions, national and global contexts, capacity, resources, environmental conditions and governance’ [6].

International and national commitments to eliminate malnutrition (especially stunting) has increased in recent years. Countries have made commitments through the sustainable development goals (SDGs) [20], the United Nations Decade of Action on Nutrition (2016-2025) [21], Malabo Declaration [22] and the Scaling Up Nutrition Movement (SUN) [23], to address malnutrition especially with focus on stunting reduction in children under five years. Similarly, World Health Assembly (WHA) targeted to reduce 40% of global stunting by 2025 [24].

Ethiopia also set stunting reduction as one goal of the Growth and Transformation Plan (GTP) with national nutrition strategies, different nutrition specific and nutrition sensitive intervention activities with focus on improving Infant and Young Child Feeding (IYCF), management of severe acute malnutrition and prevention and treatment of micronutrient deficiencies [6, 25, 26]. The Government of Ethiopia developed the Productive Safety Net Program (PSNP) in 2004 to target chronically food insecure households aimed at enabling the rural poor facing chronic food insecurity to resist shocks, create assets, and become food self-sufficient [27, 28]. PSNP user families are experiencing improved food security, community level infrastructure development, increase asset creation and protection, increase utilization of education and health services with positive short term nutritional benefit for children [29, 30].

However, stunting across preschool children is not assessed well, particularly in relation to household food security status. Therefore, the aim of this study was to determine the prevalence of stunting and its associated factors in a study population of children from food secure and food insecure households.

## Methods

### Study setting

The study was conducted in Albuko district, northeast Ethiopia. The district consists of 16 kebeles (the smallest administrative units in Ethiopia) of which 13 are rural. It has a total population of 88,954 of which 47,613 (53.5%) were male and 41,340 (46.5%) female. Children aged 24 to 59 months comprised 7,552 (8.5%) of the total [31]. According to the district early warning and response cluster report 2016, out of the total 20,483 households, 45% of them were Productive Safety Net Program (PSNP) beneficiaries [32].

There are 4 health centers and 16 health posts in the district. Albuko district is one of the districts of the Amhara region, where the routine community based nutrition intervention program is being implemented with due collaboration with Save the Children and Concern Worldwide Ethiopia [31].

Albuko is one of the most food insecure district in Amhara region. Seasonal variation of rainfall and infertile land are the main contributing factors to food insecurity. According to the 2016 district report, there were 20,483 households of which 11,183 (55%) were food secure, whereas the rest, 9,300 (45%) households (HHs) were food insecure ones. Both chronically and acutely food insecure HHs were enrolled in the PSNP. Accordingly, 95% of the HHs were chronically food insecure and 5% were acutely food insecure. The PSNP has two delivery modalities, the cash which was given to households with able bodied members who can repay it by engaging in any developmental activities, like water and soil conservation, road construction, etc. and was given to 85% of the HHs. On the other hand, the food aid was given to households with no able-bodied members and was given to 15% of the HHs [33].

### Study design and participants

A community-based comparative cross-sectional study was conducted from March to April 2017, to determine the level of stunting and tis associated factors. All preschool children aged 24-59 months who had lived at least for six months in the selected HHs were included.

### Sample size determination and sampling procedure

As it was a comparative cross-sectional study, the minimum sample size was determined by using the double population proportion formula with the following assumptions that two groups were considered based on their PSNP beneficiary status. Group one was with PSNP as exposed (as food insecure HHs) and group two was without PSNP as not exposed (food secure HHs). To estimate the minimum sample size of the study a stunting prevalence of 37.5% was used from the findings of a previous study in East Gojjam Zone among food secured households and in food surplus areas [34] and a 50% prevalence of stunting among food insecure households (since there were no previous specific studies conducted among food insecure households or areas at least to the time of the proposal stage of this work) were used. Then, the sample size estimation was calculated using Epi Info software and with a 95% confidence level and a 5% margin of error yielded 496. Considering non-response rate of 10% and a design effect of 2 for both comparative groups, a minimum sample size of 1090 was obtained.

Both multi-stage and systematic sampling techniques were used to include respondents with their preschool children. In the first step, five among 16 kebeles were selected by simple random sampling method. In the second step, sampling fraction (k) was calculated using the total number of children available in the kebeles. Households in the selected five Kebeles were stratified according to their PSNP status. Finally, after allocation proportional to population size of each kebele, the households were selected by systematic random sampling technique. Children’s ccaretakers or mothers in the selected households were interviewed and measurements of anthropometric data (height and age) were taken. As five households (5 mother-child pairs) could not be traced even after two more/repeated visits, their five adjacent counterparts were considered.

### Data collection tools and procedure

Quantitative data collection techniques/instruments were employed to gather the necessary information. A structured questionnaire composed of socio-demographic characteristics, maternal and child health characteristics, vitamin A, deworming supplementations, feeding practices of children and water hygiene and sanitation information were used to collect the data. Initially, the questionnaire was prepared in English language. Then it was translated to the local language Amharic. The daily data collection was closely supervised by the principal investigator. The questionnaire was pretested on 5% of the total samples outside the study area. During the pretest, acceptability and applicability of procedures and tools were evaluated. Six diploma graduate clinical nurses recruited as data collectors supervised by three-degree graduate clinical nurses after three days’ training given to both groups. During home visits, if the caregiver was absent, a second visit was made.

### Anthropometric measurements

Height was measured using the standardized vertical seca 213 portable stadiometer measurement.

Thick socks, shoes, and jackets or any bulky clothing of a child were removed. A child has stood with his/her back against the measuring surface with feet together flat on the floor, arms at side and knees and back straight. Head, heels, buttocks and shoulder blades of a child were touched the measuring surface. A child was looking straight ahead, then the headboard was slid gently down to the head of the child, compressing the hair. When measurer’s eyes level with the indicator, the height of the child was read to the nearest 0.1 centimetre. Then, the procedure was repeated for the second time completely. Comparison was made between the two measurements. Accordingly, if the difference between the two measurement readings were within 0.1 centimetre, the second measurement was recorded. Otherwise the average of the two measurements were taken.

When documents such as vaccination cards were available, they were used to determine the age of the children. In the absence of documentation, a local seasonal calendar method was used by the team as they were trained on how to assess age of the children. In this regard, for 941 (90.6%) children the exact age was found from the immunization card registry (family folder), for the rest, 98 (9.4%), age was approximated. Anthropometric related data of a child were transferred to the ENA/SMART software version 2012 and the Z-score of index, Height-for-Age Z-score (HAZ), was calculated using the WHO Multicenter Growth Reference Standard. The child was classified as stunted if his/her z score was less than −2SD and not stunted if Z score was ≥ − 2SD [35].

### Assessment of dietary diversity

The dietary diversity of children was assessed whether they had eaten the different food groups from yesterday’s sun rise to today’s sun rise (24hours recall method) prior to the survey date according to their mothers or caregiver’s responses. Then based on reports of their mothers or caregivers, food items consumed by the children were grouped in to seven food groups. The seven food groups were starchy staples (grains, roots, and tubers), legumes, nuts and seeds, vitamin-A rich fruits and vegetables, other fruits and vegetables, egg, dairy products (milk, yoghurt, and cheese); and flesh foods (meat, fish, poultry, and organ meats). Finally, dietary diversity score of children was calculated out of the seven food groups. A child with a DDS of four and above was classified as having good dietary diversity, otherwise classified as poor [36].

### Data quality assurance

The maximum efforts were made to maintain data quality. During the development of data collection tool, all authors reviewed the data collection instruments and the pretest was performed in similar communities before using it for the actual data collection. Careful selection and recruitment of experienced data collectors and supervisors were employed. Training was conducted for three days on survey objectives, survey methodology, measurement, and recording of anthropometric measurements (height and age), basic nutrition index, and its use. Theoretical sessions and practical demonstrations on children’s height measurement, age estimation, and collection of other nutrition related information was provided on the first two days. The third day was spared for pretesting of the questionnaire and the methodology. This consisted of field practice, including height measurement and age estimation exercise in a nearby village that had not been selected for the actual study. During the three days training, interview ethics and techniques were included in order to improve the quality of data. For pretesting, all data collectors and supervisors were participated. In addition, a strong daily field supervision was carried out to monitor the performance of data collectors and to deliver on the spot correction/feedback on any mistakes noted and to facilitate supervisor reviews of the questionnaire to ensure its completeness before the household was left.

### Data Processing and Analysis

The collected data were coded, cleaned and entered into Epi data version 3.1 and exported to Statistical Package for Social Science (SPSS) version 20 software for analysis. The WHO Anthro software was used to enter and determine the prevalence of stunting among preschool children aged 24-59 months. Height and age data were used to calculate height-for-age z score and to categorize the severity level of stunting based on the World Health Organization’s Anthro reference. Stunting is defined as low height-for-age at less than -2 SD and severe at less than -3 SD of the median value of the WHO international growth reference [35]. Prior to data analysis, missing values were checked and corrected by referring to the original data filled in the questionnaire. Descriptive statistics were used to summarize study variables. In addition, tables and figures were used to summarize the frequencies and proportions of the variables. Three models were run to identify factors associated with stunting among preschool children from food secure, and insecure households; the final model was run to identify the contributing factors to stunting among the total preschool children in the study sample. The association between stunting and the independent categorical variables was investigated by using the logistic regression model (bivariable analysis). A p value of <0.2 in the bivariable analysis were considered as a variable selection criterion. Accordingly, all variables which were significant at A p value of <0.2 were entered into the multivariable analysis in order to control the possible effects of confounders. In multivariable analysis, backward selection method was used to identify factors associated with stunting and statistical significance was considered with a p value of <0.05. The adjusted odds ratio (AOR) with a 95 % confidence interval was used to assess the strength of association. Furthermore, the fitness of the logistic regression model was checked using the Hosmer and Lemeshow goodness of fit-test, and found a p value of >0.05 for the three models fitted separately.

### Ethical consideration

Before data collection, ethical clearance was collected from the University of Gondar Institutional Ethical Review Board. The Zonal health department and the Albuko district health office gave us permission to proceed with the data collection. After informing about the whole purpose, the benefits, the risks, the confidentiality of information, and the voluntary nature of participation in the study, oral informed consent and thumb print were obtained from each participant (mother or caregiver). Names of the respondents were not recorded. Data were accessed only by the investigator to ensure confidentiality. Respondents were informed that they had the right to refuse or stop at any point of the interview. Children with nutritional and health problems were counseled and linked to cluster health facilities.

## Results

### Socio-demographic characteristics

The present study included 1073 preschool children. Out of these 3.1% preschool children HAZ index were flagged and excluded from the study. One thousand thirty-nine (1039) children were included for the final analysis. Respondents from food secure and food insecure households comprised 50.7% and 49.3%, respectively.

The mean age (±SD) of preschool children from food secure and food insecure households were 40.78 ± 10.37 and 41.01 ± 10.39 months. More than half (55.6%) of them were females. More than half (57.9%) of female preschool children were from food secure households and 53.3% female preschool children were from food insecure households. The majority of the respondents (90.8%) were housewives and slightly more than half of them (53.5) had more than four family members (Table 1).

**Table 1:** Socio-economic and demographic characteristics of the respondents from food secure and food insecure households of Albuko district, northeast Ethiopia, 2017

Characteristics	Food secure HHs *n*=527		Food insecure HHs *n*=512		Total *n*=1039	
	Frequency	Percent	Frequency	Percent	Frequency	Percent
Ethnicity						
Amhara	505	95.8	507	99.0	1012	97.4
Others^a^	22	4.2	5	1.0	27	2.6
Religion						
Muslim	493	93.5	497	97.1	990	95.3
Orthodox	34	6.5	15	2.9	49	4.7
Marital Status						
Single	32	6.1	26	5.1	58	5.6
Married	460	87.3	479	93.6	939	90.4
Others^b^	35	6.6	7	1.4	42	4.0
Under five children						
One	464	88.0	410	80.1	874	84.1
Two	58	11.0	102	19.9	160	15.4
Three	5	.9	0	0	5	.5
Mother education						
Uneducated	326	61.9	299	58.4	625	60.2
Primary	147	27.9	170	33.2	317	30.5
Secondary and above	54	10.2	43	8.4	97	9.3
Mother occupation						
Housewife	464	88.0	479	93.6	943	90.8
Government	17	3.2	6	1.2	23	2.2
Others^c^	46	8.7	27	5.3	73	7.0
Money handlers						
Husband	369	70.0	395	77.1	764	73.5
Wife	13	2.5	26	5.1	39	3.8
Both	145	27.5	91	17.8	236	22.7
Food sources						
Farming	461	87.5	486	94.9	947	91.1
Purchasing	66	12.5	26	5.1	92	8.9
Child sex						
Female	305	57.9	273	53.3	578	55.6
Male	222	42.1	239	46.7	461	44.4
Mother age						
15-34	365	69.3	244	47.7	609	58.6
35-49	161	30.6	268	52.3	429	41.3
>50	1	.2	0	0	1	.1
Child age						
24-35	221	41.9	230	44.9	451	43.4
36-47	191	36.2	167	32.6	358	34.5
48-59	115	21.8	115	22.5	230	22.1
Child birth order						
1-2	197	37.4	72	14.1	269	25.9
3-4	289	54.8	386	75.4	675	65.0
>4	41	7.8	54	10.5	95	9.1
HH family size						
<4	291	55.2	192	37.5	483	46.5
>4	236	44.8	320	62.5	556	53.5

^a^Oromo, Tigray

^b^Divorced, Windowed and Separated

^c^Trader, Student**,** Daily worker

### Maternal and child health characteristics

The majority (90.5%) and (88.3%) of the mothers had at least one Antenatal Care (ANC) visit for the index child from food secure and food insecure households and around 65.5% and 59.2% of them gave birth at health facilities, respectively. A significant proportion of preschool children (91.5%) and (90.5%) from food secure and food insecure HHs had received vitamin A and deworming supplementations during the previous six months prior to the data collection period, respectively (Table 2).

**Table 2:** Maternal and child health characteristics of the respondents from food secure and food insecure households of Albuko district, northeast Ethiopia, 2017

Characteristics	Food secure HHs *n*=527		Food insecure HHs *n*=512		Total *n*=1039	
	Frequency	Percent	Frequency	Percent	Frequency	Percent
Extra meal during pregnancy/lactation						
Yes	416	78.9	201	39.3	617	59.4
No	111	21.1	311	60.7	422	40.6
ANC visit						
Yes	477	90.5	429	83.8	906	87.2
No	50	9.5	83	16.2	133	12.8
Number of ANC						
First	13	2.7	14	3.2	27	3
Second	110	23.3	101	24	211	24
Third	164	35	148	34	312	34
Fourth and more	190	39	166	38.8	356	39
Place of delivery						
Home	182	34.5	209	40.8	391	37.6
Health facility	345	65.5	303	59.2	648	62.4
Deworming supplementation						
Yes	477	90.5	465	90.8	942	90.7
No	50	9.5	47	9.2	97	9.3
Vitamin A supplementation in the past six months						
Yes	482	91.5	459	89.6	941	90.6
No	45	8.5	53	10.4	98	9.4
Diarrheal morbidity in the past two weeks						
Yes	42	8.0	103	20.1	145	14.0
No	485	92.0	409	79.9	894	86.0

*ANC* antenatal care, *HHs* households

### Child feeding practices

Ever breast feed had been practiced by all mothers (100%) from both food secure and insecure HHs. Nearly hundred percent (98.5%) and (98%) of the mothers initiated breastfeeding timely, within an hour of delivery, respectively. Less than half (45%) of them from food secure HHs and slightly more than half (53.3%) from food insecure had started complementary feeding timely at the 6 month. The minimum dietary diversity and meal frequency score four and more among preschool children were 30.2% and 66.2% from food secure HHs, and 24% and 44.7% from food insecure ones (Table 3).

**Table 3:** Child feeding practices of the respondents from food secure and food insecure households of Albuko district, northeast Ethiopia, 2017

Characteristics	Food secure HHs *n*=527		Food insecure HHs *n*=512		Total *n*=1039	
	Frequency	Percent	Frequency	Percent	Frequency	Percent
BF initiation						
Within one hour	519	98.5	502	98.0	1021	98.3
More than one hour	8	1.5	10	2.0	18	1.7
Pre-lacteal use						
Yes	79	15.0	83	16.2	162	15.6
No	448	85.0	429	83.8	877	84.4
Pre-lacteal food type						
Milk	13	16.4	0	0	13	8.2
Water	31	39.2	43	52	74	45.6
Sugar	35	44.4	39	47.9	74	45.6
Honey	0	0	1	0.1	1	0.6
Colostrum use						
Yes	483	91.7	493	96.3	976	93.9
No	44	8.3	19	3.7	63	6.1
CF initiation						
Below six month	92	17.5	80	15.6	172	16.6
At six month	237	45.0	273	53.3	510	49.1
After six month	198	37.6	159	31.1	357	34.4
Feeding method						
Bottle	110	20.9	112	21.9	222	21.4
Cup	231	43.8	213	41.6	444	42.7
Hand	169	32.1	174	34.0	343	33.0
Others^a^	17	3.2	13	2.5	30	2.9
Dietary diversity						
<4 food groups	368	69.8	389	76.0	757	72.9
>4 food groups	159	30.2	123	24.0	282	27.1
Meal frequency						
<4 times	178	33.8	283	55.3	461	44.4
>4 times	349	66.2	229	44.7	578	55.6
Types of food						
Injera	57	10.8	56	10.9	113	10.9
Milk	107	20.3	99	19.3	206	19.8
Porridge	214	40.6	257	50.2	471	45.3
Smashed potato	149	28.3	100	19.5	249	24.0

*BF* Breast feeding, *CF* Complementary feeding

^a^spoon, Dietary diversity: number of food groups consumed by an individual

### Water hygiene and Sanitation Characteristics

The findings showed that 90.5% of food secure households and 86.1% of food insecure households source their drinking water from protected sources such as protected spring, pipe, protected well etc. However, 9.5% and 13.9% of food secure and food insecure households source their drinking water from unprotected sources such as river, unprotected spring and open wells (Table 4).

**Table 4:** Water source, sanitation and hygienic characteristics of the respondents from food secure and food insecure households of Albuko district, northeast Ethiopia, 2017

Characteristics	Food secure HHs *n*=527		Food insecure HHs *n*=512		Total *n*=1039	
	Frequency	Percent	Frequency	Percent	Frequency	Percent
Sources of water						
Protected source	477	90.5	441	86.1	918	88.4
Unprotected source	50	9.5	71	13.9	121	11.6
Amount of water used (litter)						
<40	201	38.1	194	37.9	395	38.0
>40	326	61.8	318	62.1	644	61.9
Time spent to water (minutes)						
<30	363	68.9	346	67.6	709	68.2
>30	164	31.1	166	32.4	330	31.8
Latrine availability						
Yes	413	78.4	476	93.0	889	85.6
No	114	21.6	36	7.0	150	14.4
Types of latrine						
Pit	409	99.1	464	97.4	873	98.2
Flush	0	0	2	0.4	2	0.2
Ventilated improved	4	0.9	10	2.2	14	1.6

*HHs* households

Time spent to water: the time it takes to reach to water source (for single trip)

### Prevalence of stunting

The overall prevalence of stunting among preschool children was 39.3% (95%CI: 36.3%-42.3%), while that of such children from food insecure and food secure households was 42.8% (95%CI: 38.4%-47.2%) and 35.9% (95%CI: 31.7%-40.1%), respectively. The proportion of severely stunted from food insecure households was 25.2% and that of food secure was 18.6%. A high proportion of stunting (46.6%) was observed among male preschool children as opposed to 33.4% among females in the whole study sample. There was an increasing trend of stunting across age categories among children from food secure households (FSHHs). The highest prevalence of stunting (40.3%) was observed in the 48-59-month age group and the lowest prevalence (28.8%) in the 24-35-month age group. Conversely, the trend of stunting among children from food insecure households (FIHHs) was decreased as the age of children increased. Children 24-35-month old were at higher odds of stunting than children 48-59 month (Table 5).

**Table 5:** Prevalence of stunting among preschool children from food secure and food insecure households of Albuko district, northeast Ethiopia, 2017

Characteristics	Food secure(*n*=527)			Food insecure(*n*=512)			Total (*n*=1039)		
	Number	Height-for-age (%)		Number	Height-for-age (%)		Number	Height-for-age (%)	
		% < -3SD	% < -2SD		% < -3SD	% < -2SD		% < -3SD	% < -2SD
Age									
24-35	132	17.4	28.8	137	32.8	51.8	269	25.3	40.5
36-47	209	16.7	36.4	183	22.4	41	392	19.4	38.5
48-59	186	21.5	40.3	192	22.4	38	378	22	39.2
Sex									
Male	222	23.9	42.8	239	28.9	50.2	461	26.5	46.6
Female	305	14.8	30.8	273	22	36.3	578	18.2	33.4
Total	527	18.6	35.9	512	25.2	42.8	1039	21.8	39.3

*SD* standard deviation

### Factors associated with stunting among preschool children from food secure HHs

In both the bivariable and multivariable analysis, mother’s education, family size, child sex, child birth order and the amount of water used were found to be significantly associated with stunting among preschool children from food secure households. Accordingly, preschool children having uneducated mothers and primary school complete were 5.2 and 4.2 times more likely to be stunted than preschool children whose mothers had higher education (secondary and above) (AOR= 5.24, 95% CI; 2.30-11.91) and (AOR= 4.2, 95% CI; 1.77-9.97), respectively. Likewise, increased odds of stunting were observed among children from four and more family member households (AOR= 4.33, 95% CI; 2.92-6.41) and children in fourth and above birth order (AOR= 2.25, 95% CI; 1.03-4.92). In addition, male sex increased the likelihood of developing stunting 1.67 more times (AOR= 1.67, 95% CI; 1.13-2.47) (Table 6).

**Table 6:** Factors Associated with stunting among food secure households in preschool children of Albuko district, northeast Ethiopia, 2017 (*n*= 527)

Characteristics	Stunting		COR (95%)	AOR (95%)
	Yes (%)	No (%)		
Mother education				
Uneducated	129 (39.6)	197 (60.4)	3.77 (1.721-8.24)	5.24 (2.30-11.91) ***
Primary	52 (35)	95 (65)	3.15 (1.38-7.17)	4.200 (1.77-9.97) ***
Secondary and above	8 (14.8)	46 (85.2)	1	1
Family size				
<4	65 (22.3)	226 (77.7)	1	1
>4	124 (52.5)	112 (47.5)	3.85 (2.64-5.61)	4.33 (2.92-6.41) ***
Extra food during pregnancy/lactation				
Yes	138 (33)	278 (67)	1	1
No	51 (45.9)	60 (54.1)	1.71 (1.12-2.62)	1.19 (0.72-1.95)
Child sex				
Female	94 (30.8)	211 (69.2)	1	1
Male	95 (42.7)	127 (57.3)	1.68 (1.17-2.41)	1.67 (1.13-2.47) **
Child birth order				
1-2	51 (25.8)	146 (74.2)	1	1
3-4	114 (39.4)	175 (60.6)	1.87 (1.25-2.77)	1.14 (0.72-1.80)
>4	24 (58.5)	17 (41.5)	4.04 (2.01-8.13)	2.25 (1.03-4.92) *
Amount of water used (litter)				
<40	84 (41.7)	117 (58.3)	1.51 (1.05-2.18)	1.58 (1.06-2.34) *
>40	105 (32.2)	221 (67.8)	1	1
Time spent to water (minutes)				
<30	123 (33.8)	240 (66.2)	1	1
>30	66 (40.2)	98 (59.8)	1.31 (0.90-1.92)	1.29 (0.84-1.98)

Hosmer-Lemeshow test p value=0.74

Time spent to water; the time it takes to reach to water source (for single trip)

(1.00=Reference ***=p<0.001 ** =p<0.01 *= p<0.05)

### Factors associated with stunting among preschool children from food insecure HHs

In the multivariable analysis, level of maternal education, family size, extra meal consumption during pregnancy/lactation, child sex, child age, and dietary diversity score were significantly associated with stunting among preschool children from food insecure HHs.

The risk of being stunted was 3 times more likely among preschool children from uneducated mothers compared to preschool children from educated mothers (secondary and above education) (AOR= 3.07, 95%CI; 1.40-6.75). Preschool children in households of four and more family members, (AOR= 3.64, 95% CI; 2.37-5.58) male sex (AOR= 1.73, 95% CI; 1.16, 2.58), and 24-36-month age group (AOR= 2.37, 95% CI; 1.41, 3.99) were significantly associated with stunting (Table 7).

**Table 7:** Associated factors of stunting among preschool children from food insecure households of Albuko district, northeast Ethiopia, 2017 (*n* = 512)

Characteristics	Stunting		COR (95%)	AOR (95%)
	Yes (%)	No (%)		
Mother education				
Uneducated	160 (53.6)	139 (46.4)	3.80 (1.81-7.99)	3.08 (1.40-6.76) **
Primary	49 (28.9)	121 (71.1)	1.34 (0.61-2.92)	0.97 (0.42-2.22)
Secondary and above	10 (23.3)	33 (76.7)	1	1
Family size				
<4	50 (26.1)	142 (73.9)	1	1
>4	169 (52.8)	151 (47.2)	3.18 (2.15-4.69)	3.64 (2.37-5.58) ***
Extra food during pregnancy/lactation				
Yes	72 (35.8)	129 (64.2)	1	1
No	147 (47.3)	164 (52.7)	1.61 (1.12-2.31)	1.71 (1.14-2.58) **
Child sex				
Female	99 (36.3)	174 (63.7)	1	1
Male	120 (50.2)	119 (49.8)	1.77 (1.24-2.53)	1.73 (1.17-2.58) **
Child age				
24-35	119 (51.8)	111 (48.2)	2.26 (1.41-3.61)	2.37 (1.41-3.99) ***
36-47	63 (37.7)	104 (62.3)	1.28 (0.77-2.11)	1.47 (0.84-2.58)
49-59	37 (32.2)	78 (67.8)	1	1
Dietary diversity score				
<4 food groups	180 (46.3)	209 (53.7)	1.86 (1.21-2.85)	1.92 (1.20-3.09) **
>4 food groups	39 (31.8)	84 (68.2)	1	1
Meal frequency/day				
<4 times	132 (46.7)	151 (53.3)	1.43 (1.00-2.03)	1.18 (0.78-1.80)
>4 times	87 (38)	142 (62)	1	1

Hosmer-Lemeshow test p value=0.37

Dietary diversity score: the sum total of food groups consumed by an individual in the past 24 hours

(1.00=Reference ***=p<0.001 ** =p<0.01 *= p<0.05)

### Factors associated with stunting among preschool children (whole sample population)

The present study revealed that, maternal education, family size, sex of child, and extra meal consumption during pregnancy/lactation were found to be significantly associated with stunting among preschool children during the multivariable analysis in the final model.

The risk of being stunted for preschool children whose mothers were uneducated and primary school level were 4.38 and 2.1 times more than in preschool children whose mothers had secondary and above education (AOR= 4.38, 95% CI; 2.51, 7.66) and (AOR= 2.10, 95% CI; 1.17, 3.79). Preschool children in households of four and more members were 3.4 times more likely to be stunted compared to children in less than four family members (AOR= 3.47, 95% CI; 2.62, 4.60). Furthermore, the analysis of logistic regression revealed that male sex increased the likelihood of stunting by 1.71 times compared to female sex (AOR= 1.71, 95% CI; 1.30, 2.24) (Table 8).

**Table 8:** Associated factors of stunting among preschool children from FSHHs and FIHHs of Albuko district, northeast Ethiopia, 2017 (*n*=1039)

Characteristics	Stunting		COR (95%)	AOR (95%)
	Yes (%)	No (%)		
Mother education				
Uneducated	289 (46.3)	336 (53.7)	3.78 (2.21-6.45)	4.38 (2.51-7.66) ***
Primary	101 (31.9)	216 (68.1)	2.05 (1.17-3.61)	2.11 (1.17-3.79) *
Secondary and above	18 (18.6)	79 (81.4)	1	1
HHFS status				
Secure	219 (40.3)	324 (59.7)	1	1
Insecure	189 (38.1)	307 (61.9)	1.10 (0.86-1.41)	1.09 (0.84-1.44)
Family size				
<4	115 (23.8)	368 (76.2)	1	1
>4	293 (52.7)	263 (47.3)	3.565 (2.73-4.66)	3.47 (2.62-4.60) ***
Extra food during pregnancy/lactation				
Yes	198 (47)	224 (53)	1	1
No	210 (34.1)	407 (65.9)	1.71 (1.33-2.21)	1.44 (1.09-1.90) **
Child sex				
Female	193 (33.4)	385 (66.6)	1	1
Male	215 (46.7)	246 (53.3)	1.74 (1.36-2.24)	1.71 (1.30-2.24) ***
Child age				
24-35	192 (42.6)	259 (57.4)	1.39 (1.00-1.93)	1.33 (0.93-1.91)
36-47	136 (38)	222 (62)	1.15 (0.81-1.62)	1.151 (0.79-1.68)
48-59	80 (34.8)	150 (65.2)	1	1
Dietary diversity				
<4 food groups	313 (41.4)	444 (58.6)	1.39 (1.04-1.85)	1.34 (0.98-1.83)
>4 food groups	95 (33.7)	187 (66.3)	1	1
Meal frequency/day				
<4 times	202 (43.9)	259 (56.1)	1.41 (1.10-1.81)	1.03 (0.77-1.38)
>4 times	206 (35.6)	372 (64.4)	1	1
Time spent to water				
<30 minutes	266 (37.6)	443 (62.4)	1	1
>30 minutes	142 (43.1)	188 (56.9)	1.26 (0.96-1.64)	1.23 (0.92-1.64)

Hosmer-Lemeshow test p value=0.55

HHFS; Household Food Security status

Time spent to water: the time it takes to reach to water source (for a single trip)

(1.00=Reference ***=p<0.001 ** =p<0.01 *= p<0.05)

## Discussion

The result of the present study showed that the prevalence of stunting among preschool children from both food secure and food insecure households in the community was high which clearly confirming that currently chronic malnutrition (stunting) is a widespread public health problem significance in the study area. Poor breastfeeding practices, poor complementary feeding, low level health seeking behaviours, close birth spacing, low parental education, and low wealth status (poor socio-economic status) are some of the factors which precipitate the problem [37].

The prevalence of stunting was found to be high among preschool children from both food secure and food insecure households (35.9%, 95%CI; 31.7, 40.1) and (42.8%, 95%CI; 38.4, 47.2) respectively. Of these, 18.6% preschool children from FSHHs and 25.2% from FIHHs were severely stunted.

The findings of this study were consistent with those of other local studies from East and West Gojjam, Ethiopia which reported a stunting prevalence of 37.5% and 38.3% among children from food secure and food insecure households (FSHHs and FIHHs), respectively and reported no significant variations among both groups [34]. This similarity may be due to contextual similarities in socio- demographic and economic characteristics, and feeding pattern of children of the study areas.

On the other hand, the level of stunting among food insecure HHs (42.8%) and food secure HHs (35.9%) in the present study was lower than those of studies conducted in other parts of Ethiopia. For example, Tigray reported 52% and 46.1% prevalence of stunting among children from FIHHs and FSHHs, respectively [38], and Shewa, reported 54.2% and 46.7% among children from FIHHs and FSHHs, respectively [39]. The possible reason for these discrepancies might be the fact that as Albuko is one of the districts in which community based nutrition intervention has been implemented for a long period of time which in turn expose the community to be aware of nutritional interventions thereby it has contributing to the reduction of stunting and other forms of malnutrition in the study are. Additionally, there were high maternal illiteracy rate and most of the mothers were housewives among the studies done in Tigray and Shewa of Ethiopia. The overall prevalence of stunting in this study was 39.3% (95% CI:36.3, 42.3). The prevalence of severe stunting was 21.8%. The finding of this study was consistent with the national prevalence of stunting (38%, EDHS report, 2016) for children under five years [8]. Again, the current finding was also in agreement with that of a baseline survey report for Infant and Young Child Feeding Program 2014, conducted in Albuko district, Ethiopia, by Alive and Thrive and reported a stunting prevalence of 38% [40]. However, the overall stunting prevalence (39.3%) of the present study was lower than that of Amhara Region (46%), Ethiopia [8].

Conversely, this study showed a higher stunting prevalence compared with that of Ethiopian Somali Region reported a stunting prevalence of 22.9% [41] and Gambella, Ethiopia reported 33.7% of prevalence [42]. The possible reason for the occurrence of the difference between the current and previous studies may be arisen from the age groups of study subjects included. For the present study, children between the age group of 24-59 months were included while the previous studies included children less than 24 months. Stunting for children under age 5 sharply increases between age 6 and 23 months, and peaks at age 24-35 months [8]. Hence, the prevalence of stunting in this study might be overestimated by the inclusion of older children as compared to those studies. Most of the mothers in this study were uneducated and housewives. Higher odds of stunting was observed among children whose mothers were illiterate [43, 44].

In the present study, mother’s education, family size, child sex were factors associated with stunting among preschool children from both FSHHs and FIHHs during multivariable analysis which was analyzed separately. Child birth order and the amount of water (<30 litters) used per day showed an associated with stunted children from food secure households, whereas, extra meal during pregnancy/lactation, child age, and low dietary diversity score of less than the WHO recommendation were predictors of stunting in children from food insecure households.

In the overall model among the variables fitted to the multivariable analysis, only four independent variables, namely maternal education, family size, extra meal taking during pregnancy or lactation, and child sex remained positively associated with child stunting, whereas household food security status, age of the child, and child’s dietary diversity did not show an association with child stunting. The odds of stunting were higher among children from uneducated mothers than educated mothers 4.38 (AOR = 4.38, 95% CI: 2.51-7.66). The finding was parallel with EDHS 2016 report which revealed that higher stunting rate (42%) was observed among children who were from illiterate mothers as compared to those children who were from literate mothers (17%) [8]. Similar research findings in Ethiopia were also reported by other studies which tried to show mothers’ education was emerged as the key predictor of stunting [34, 45]. It is argued that educated mothers have higher nutritional knowledge which intern contributed to child feeding practices of mothers and finally helped them to have a well-nourished children but, mothers with no formal education were do not [45].

Children from four and above family members were more likely to be stunted as compared to children from less than four family members. Studies from Tigray Region, Ethiopia [38] and India [46] reported similar results. The association between large family size and stunting among preschool children can be partially explained by as the number of families increased in the household, the food consumption and expenditure will also be increased. As a result, the probability of children getting adequate nutrition and energy will be less despite the fact that children needs more energy and micronutrient to support their rapid growth and development. Hence, unable to supply the necessary energy and micronutrient requirements by children for a prolonged period eventually leads to development of manifestation of chronic malnutrition or stunting. Furthermore, Families with more children generally devote less time to care their children [37, 47].

The habits of taking extra meals during pregnancy or lactation was also an associated factor for stunting in that children born to mothers who did not take extra meals during pregnancy or lactation were 1.44 times more likely to be stunted than children of mothers who had taken extra meals during pregnancy or lactation (AOR = 1.437, 95% CI: 1.089-1.895). This is true as women of reproductive age are especially vulnerable to chronic energy deficiency because they have a high nutrient needs and lack of adequate nutrients during pregnancy and breast feeding put the baby at a higher risk of being stunted [8]. But it should be noted that taking extra meal was measured by asking the mother’s recall for the previous pregnancy or lactation period for the study child. As a result, it cannot be measured and difficult to ascertain whether it was taken during pregnancy, lactation or during both periods by the mother.

Stunting prevalence cross sectional studies in Ethiopia [43, 44] , EDHS 2016 report [8], and from Sub Saharan Africa [48, 49] reported that the prevalence of stunting among under five male children was consistently higher than among under five female children. The present study also found a similar result that the odds of being stunted were 1.71 times higher among under five male children than among under five female children. (AOR = 1.708, 95% CI: 1.302-2.240). This result could be due to unmeasured factors like parental gender preference or sex difference (perceived higher needs for boys) in feeding practice. In addition to behavioral differences, there might be biological differences. However, up to today mechanisms underlying sex difference in stunting prevalence are poorly understood [50].

In the multivariable logistic regression analysis of the final model, ANC visits and availability of latrine were among the factors that were not associated with stunting. The absence of association between ANC visits and stunting may be partially explained by there was no difference in ANC visits between mothers from FSHHs and FIHHs. Information on ANC visits was collected from the mother’s recall for the previous pregnancy for the study child. However, all of these information given by mother may not be belonging to the study child (if the study child was not the previous pregnancy) due to the fact that the mother might not be able to remember or differentiate for which her previous pregnancy or child she had followed an ANC. As a result, the mother may report her ANC visits for the younger siblings rather than for the study child.

Greater FIHHs had a latrine than their counterparts. This may be due to the PSNP enforces the beneficiaries to construct more latrines and the difference in educational level of the mothers as more educated mothers were from FIHHs.

The current study finding revealed out no difference was observed among under five children as being from Productive Safety Net Program (PSNP) beneficiaries or FIHHs and non-beneficiaries (FSHHs) in terms of their stunting status. that the two comparative groups were not different significantly. According to this study finding, being a food secured household did not show any association with child stunting. This finding was in line with study reports from in Ethiopian and Brazil which showed the absence of association [34, 51]. The absence of association might be because of other factors that directly or indirectly affect the nutritional status of children [52]. The absence of association in this study may further be explanation by the presence of an opposite trend of stunting prevalence across children’s age groups in FSHHs and FIHHs. Less number of stunted children and high proportion of stunted children were noticed among the youngest and older age categories in FSHHs respectively. While the rate of stunting was higher in the youngest and its rate reduced in the older age categories in FIHHs. Following the opposite trend of stunting between the two groups the association might be masked when the two groups analysed together in the overall model analysis. Furthermore, community based nutrition, IYCF, National Nutrition Program (NNP) and water, sanitation and hygiene interventions which are currently implemented in the study area along with the PSNP can also be mentioned as a reason for the absence of significant association between household food security status and child stunting status.

### Limitation of the study

The current study has addressed its objectives to determine the prevalence of stunting and factors associated with it among preschool children aged 24–59 months in the Rural community of Albuko district, northeast Ethiopia. Due to a cross-sectional design, it was difficult to examine any potential temporal relationships. Since the study included children aged 24-59 months there is a potential recall bias among respondents answering questions relating to events happening in the past two years and above. As for example; ANC visits, taking extra meal during pregnancy or lactation, vitamin A and deworming supplementations and dietary diversity of children in the past 24 hours. In addition, the study is not free from measurement errors during measuring height and determining age of the children although a pre-test, instrument calibration, training of data collectors and close supervisions during data collection were carried out to minimize bias. Finally, information on some important confounding variables such as maternal nutrition status, minimum dietary diversity for women, hand washing practice among mothers and other forms of undernutrition (wasting and underweight) among preschool children were not collected.

## Conclusion

Based on the findings of the current study, it can be concluded that the rate of stunting was high among preschool children in the study area confirming that stunting still remains a public health problem in Albuko district even though it is declining from 44% in 2011 to 38% in 2016 among under five children [7, 8]. Though PSNP is a proven strategy in reducing the burden of childhood stunting, this study showed that there is no significant variation in the magnitude of stunting. However, it does not mean that PSNP intervention is not important in reducing the burden of stunting. Furthermore, the etiology of stunting is multifactorial and thus food security cannot capture all factors at work. Therefore, enhancing maternal education, maternal nutrition and utilization of family planning services are some of the critical interventions to curb chronic nutrition problems among preschool children.

## Abbreviations

ANC: Ante Natal Care
AOR: Adjusted Odds Ratio
CI: Confidence Interval
COR: Crude Odds Ratio
DDS: Dietary Diversity Score
EDHS: Ethiopian Demographic Health Survey
ENA: Emergency Nutrition Assessment
FANTA: Food and nutrition technical assistant
FAO: Food and Agricultural Organization
FIHHs: Food insecure households
FSHHs: Food secure households
GTP: Growth and Transformation Plan
HAZ: Height for age Z-score
HHFS: Household food security
HHs: Households
IYCF: Infant and young child feeding
NNP: National Nutrition Program
PSNP: Productive Safety Net Program
SD: Standard Deviation
SDGs: Sustainable Development Goals
SPSS: Statistical Package for Social Sciences
SUN: Scaling Up Nutrition
UNCEF: United Nation Children’s Fund
WHA: World Health Assembly
WHO: World Health Organization
